# Apoptosis inducers in chronic lymphocytic leukemia

**DOI:** 10.18632/oncotarget.1480

**Published:** 2013-11-19

**Authors:** Christian Billard

**Affiliations:** ^1^ INSERM U 872, Centre de Recherche des Cordeliers, Equipe 18, Paris, France; ^2^ Université Pierre et Marie Curie (UMRS 872), Paris, France

**Keywords:** CLL, impaired cell death program, apoptosis-targeted therapies, apoptosis inducers, Bcl-2 family proteins

## Abstract

Chronic lymphocytic leukemia (CLL) is characterized by a typical defect in apoptosis and is still an incurable disease. Numerous apoptosis inducers have been described. These synthetic compounds and natural products (mainly derived from plants) display antileukemic properties *in vitro and in vivo* and some have even been tested in the clinic in CLL. They act through several different mechanisms. Most of them involve proteins of the Bcl-2 family, which are the key regulators in triggering the mitochondrial pathway of caspase-dependent apoptosis. Thus, the Mcl-1/Noxa axis appeared as a target. Here I overview natural and synthetic apoptosis inducers and their mechanisms of action in CLL cells. Opportunities for developing novel, apoptosis-based therapeutics are presented.

## 1 INTRODUCTION

Apoptosis is a physiological cell suicide program that is essential for the regulation of development, the maintenance of homeostasis and the prevention of tumorigenesis. Evading the apoptotic program is one of the hallmarks of cancer and represents an important mechanism in clinical resistance to therapies [1,2]. This is particularly true for chronic lymphocytic leukemia (CLL), a currently incurable condition that is clearly characterized by impaired apoptosis. The development of therapeutic strategies that target apoptosis in CLL is therefore a very important issue.

### 1.1 Pathways in caspase-dependent apoptosis

The apoptotic machinery comprises two main activation pathways (the extrinsic and the intrinsic pathways) and an execution phase mediated by proteases of the caspase family [3]. The extrinsic pathway (also known as the death receptor pathway) is triggered by ligation of death receptors (TNF-R, Fas, DR4) by their respective ligands (TNF, Fas L, TRAIL) and recruitment of adapter molecules, which activates the initiator caspase-8. The intrinsic pathway (also known as the mitochondrial pathway) integrates various intracellular signals at the mitochondrial membrane and is regulated by Bcl-2 family proteins (that share at least one of the four Bcl-2 homology domains, BH1 to 4). The prosurvival members of the family (Bcl-2, Bcl-xL, Bcl-w, Mcl-1 and A1) sequester the proapoptotic members Bax and Bak. Upon stimulation, the family's BH3-only members (so-called because they only have the BH3 domain) Bim, Puma, Bid, Bad, Noxa, *etc*., bind to and antagonize the prosurvival proteins. The sequestered Bax and Bak are thus released from their prosurvival ligands and then activated. There is another activation pathway in which Bax and Bak are directly activated by certain BH3-only proteins [3]. In any event, the activation of Bax and Bak induces pore formation at the outer mitochondrial membrane. The resulting membrane permeabilization favors the cytoplasmic release of apoptogenic factors, including cytochrome *c*. The latter interacts with the adapter Apaf-1 which enables the initiator caspase-9 to be activated. In turn, caspases-8 and -9 trigger the activation of effector caspases. These proteases can be inactivated by endogenous inhibitors of apoptosis protein (IAP). The IAP antagonist Second mitochondria-derived activator of caspases (Smac) is one of the apoptogenic factors released from the mitochondria [3]. Furthermore, several signaling pathways are known to interfere indirectly with apoptosis: these include the nuclear factor-kappaB (NF-kB) pathway, which activates the transcription of antiapoptotic proteins (such as Mcl-1 and IAPs) or the phosphatidylinositol-3-kinase (PI3K)/AKT pathway that phosphorylates several protein targets of relevance to apoptosis (such as Bad) [4]. Both pathways may thus downregulate caspase-dependent apoptosis. In contrast, the p53 pathway is an integrator of cellular stress and is capable of stimulating apoptosis notably through transcriptional activation of proapoptotic proteins (e.g., Puma and Noxa).

### 1.2 Defective apoptosis in CLL

**Figure 1:**
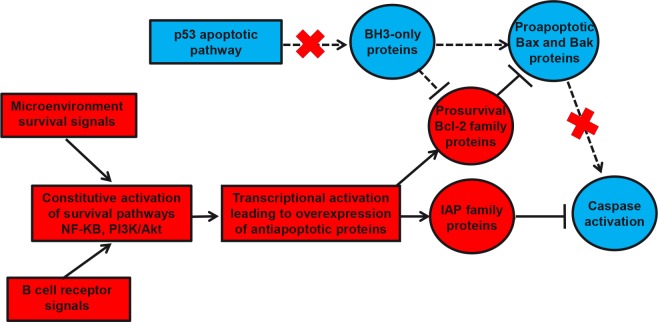
Schematic representation of the impaired mitochondrial caspase-dependent apoptosis in CLL cells Antiapoptotic factors are colored in red and proapoptotic components are colored in blue. Constitutive activation of survival pathways by microenvironment signals and B cell receptor signaling leads to the transcriptional activation of prosurvival factors from the Bcl-2 and IAP families which are thus overexpressed in CLL cells. The increased antiapoptotic activity exerted by these factors results in (i) sequestration of the proapoptotic proteins Bax and Bak (which thus prevents the mitochondrial membrane permeabilization and the subsequent cascade of caspase activation) and (ii) direct inhibition of caspase activities by IAP proteins. Moreover, deficiencies in the p53 apoptotic pathway (which are frequently observed in CLL) reduce the expression of BH3-only proteins like Puma and Noxa (known to block the antiapoptotic activity of prosurvival Bcl-2 family members and promote Bax and Bak activation). IAP, inhibitor of apoptosis protein; NF-kB, nuclear factor-kappaB; PI3K, phosphoinositol-3 kinase.

Chronic lymphocytic leukemia results from the clonal expansion of a CD5-positive subpopulation of B lymphocytes which progressively accumulate in the bone marrow, lymph nodes and peripheral blood. Although proliferative pools in the bone marrow and lymph nodes probably feed the blood compartment, the leukemic cells in the blood are quiescent but are unable to initiate their apoptotic program. This situation is due to several factors including defects in the CLL cells' apoptotic machinery and excessive survival signals delivered by the microenvironment [5–9]. Bone marrow stroma cells, nurse-like cells and T cells produce chemokines and cytokines that activate survival pathways such as NF-kB or PI3K/AKT. Indeed, these pathways are constitutively activated in CLL cells and this leads to the transcription and overexpression of key antiapoptotic proteins (notably several members of the Bcl-2 and IAP families). X-linked IAP (XIAP) appears to be the IAP that has a major role in caspase inactivation [10]. Regarding the prosurvival Bcl-2 proteins, it is now well established that in addition to Bcl-2 itself, Mcl-1 is a crucial player in impaired apoptosis in CLL cells [6,7,11,12]. Indeed, silencing Mcl-1 with small interfering RNA (siRNA) is enough to induce apoptosis in CLL cells [13]. Other signaling pathways are also involved in the overexpression of antiapoptotic proteins in CLL cells: for example, B cell receptor (BCR) signals reportedly upregulate Mcl-1 expression through the PI3K/AKT pathway [8]. Lastly, alterations in apoptosis regulators such as p53 (which are frequently observed in CLL) may be implicated in the defective apoptosis. A schematic representation of the impaired apoptotic machinery in CLL cells is shown in Figure 1.

### 1.3 Chronic lymphocytic leukemia is an incurable disease

The treatment of CLL patients with combinations of alkylating agents, purine analogues and monoclonal antibodies (fludarabine, cyclophosphamide and rituximab or allied agents) has significantly increased both the proportion and duration of complete remissions, and prolonged the overall survival [14,15]. Nevertheless, the treatment failure rate is still high and most treatment-responsive patients subsequently relapse.

### 1.4 Apoptosis-based strategies for CLL

Various ways of reactivating the apoptotic machinery in primary CLL cells have been developed over the past 15 years as extensively reviewed [5,8,16].

These approaches include inhibition of NF-kB or PI3K/AKT pathways (using for example CAL-101, a PI3Kdelta isoform), enhancement of p53-dependent apoptosis (using nutlin-3a or inhibitors of poly[ADP-ribose] polymerase-1), stimulation of the extrinsic pathway through the death receptors, interference with BCR signaling (using notably dasatinib, a Lyn kinase inhibitor and the Syk inhibitor fostamatinib) or with survival signals delivered by the leukemic microenvironment (particularly lenalidomide that repairs the T cell immunologic synapse defect, and CXCR4 targeting using plerixafor), novel chemotherapeutics (e.g. bendamustine, forodesine, acadesine) and monoclonal antibodies. However, the most extensively studied approach has been the identification of agents capable of directly triggering the intrinsic mitochondrial pathway of apoptosis. This prompted the discovery of the *in vitro* apoptosis inducers reviewed in the present article.

## 2 APOPTOSIS INDUCERS IN CLL CELLS

Apoptosis inducers comprise a broad variety of synthetic and natural products as illustrated in Tables 1 and 2. Some of these agents were found to inhibit the expression or activity of antiapoptotic molecules whereas others can upregulate proapoptotic proteins.

**Table 1:** Functional diversity of apoptosis inducers in chronic lymphocytic leukemia cells

**Inhibitors of prosurvival protein expression (Bcl-2 and IAP families)**	
Cyclin-dependent kinase inhibitors	Flavopiridol, roscovitine, dinaciclib, SNS-032
Translational inhibitors	Homoharringtonine, silvestrol
Small interfering RNA	Mcl-1 siRNA
Antisense oligonucleotides	Oblimersen
**Inhibitors of prosurvival protein activity**	
BH3 mimetics	ABT series, AT-101, ApoG2, obatoclax, 072RB
SMAC mimetics	XIAP antagonist
**Enhancers of proapoptotic protein expression**	
Proteasome inhibitors	Bortezomib, lactacystin, MG-132, carfilzomib
Plant-derived proteasome inhibitors	EGCG, quercetin, apigenin, xanthohumol
Histone deacetylase inhibitors	Depsipetide, valproic acid, MGCD0103, vorinostat
**Activators of apoptotic pathways**	
p53 activators	Nutlin-3a, PARP inhibitor
Death receptor pathway activators	TRAIL
**Inhibitors of survival pathways**	
Nuclear factor-kB inhibitors	BAY-117082
Phosphoinositol-3 kinase/AKT inhibitors	CAL-101, Akt-1/2
Inhibitors of microenvironment signals	Lenalidomide, plerixafor (anti-CXCR4)
**Modulators of other signaling pathways**	
B cell receptor signaling inhibitors	Fostamatinib (Syk), dasatinib (Lyn kinase)
Kinase inhibitors	Sorafenib (multi-kinases), imatinib (Abl kinase)
JNK activators	Fenretinide
**Others**	
Anticancer drugs and other therapeutics	Fludarabine, vinblastine, acadesine, bendamustine
Hormones and anti-inflammatory agents	Corticoids, aspirin
Cytokines	Interleukin-21
Hsp90 inhibitors	17-DMAG
Multi-target compounds	Resveratrol, curcumin and other polyphenols, triterpens, xanthones, hyperforin

SiRNA, small interfering RNA; EGCG, epigallocathechin gallate; IAP, inhibitor of apoptosis protein; BH3, Bcl-2 homology domain 3; SMAC, second mitochondria-derived activator of caspases; PARP, poly (ADP-ribose) polymerase.

**Table 2:** Structural diversity of apoptosis inducers in chronic lymphocytic leukemia cells

Type of compound			Examples
**Plant-derived compounds**		
Polyphenols	Flavonoids	Flavones	Flavopiridol, apigenin
		Flavonols	Quercetin
		Flavanols	Epigallocatechin gallate (EGCG)
		Prenylated	Xanthohumol
	Stilbenoids		Resveratrol, combretastatins
	Aldehydes		Gossypol, AT-101
	Curcuminoids		Curcumin
Vinca alkaloids			Vinblastine
Other alkaloids			Homoharringtonine, silvestrol
Phloroglucinols		Prenylated	Hyperforin
Xanthones			Allanxanthone C, macluraxanthone
**Other natural products and derivatives**			
Starfish alkaloid			Roscovitine/seliciclib
Cytokines			TRAIL, Interleukin-21
Hormones			Corticoids
Small organic molecule BH3 mimetics			Obatoclax, Apogossypolone (ApoG2)
BH3 peptide-derived BH3 mimetics			072RB
Bacteria-derived products			Lactacystin, depsipeptide
**Synthetic compounds**			
Chemical inhibitors		Proteasome	Bortezomib
		HDAC	MGCD0103
		CDK	Dinaciclib, SNS-032
		Other kinases	Dasatinib, fostamatinib, sorafenib
		Signaling pathways	CAL-101, nutlin-3
		Microenvironment	Lenalidomide, plerixafor
Terpenoids		Triterpens	CDDO
Small interfering RNA			Mcl-1 siRNA
Antisense oligonucleotides			Oblimersen (anti-Bcl-2)
Anticancer drugs and other therapeutics			Fludarabine, bendamustine, forodesine
Retinoid derivatives			Fenretinide
Synthetic BH3 mimetics			ABT series

BH3, Bcl-2 homology domain 3; HDAC: histone deacetylase; CDK: cyclin-dependent kinase; CDDO: 2-cyano-3,12-dioxoolean-1,9-dien-28oic acid.

### 2.1 Inhibitors of antiapoptotic protein expression

#### Flavopiridol

**Figure 2:**
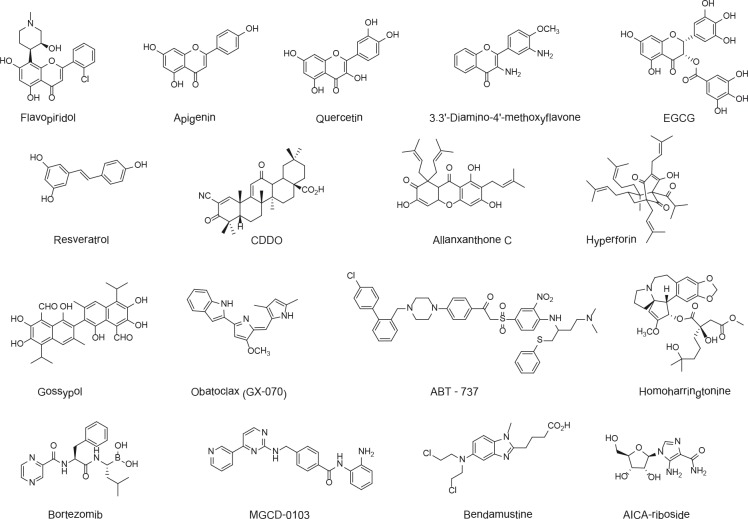
Chemical structures of some apoptosis inducers in CLL EGCG, epigallocatechin gallate; CDDO, 2-cyano-3,12-dioxoolean-1,9-dien-28oic acid.

Flavopiridol is a semisynthetic favone derived from rohitukine (an alkaloid isolated from Indian plants). Its chemical structure is shown in Figure 2. To the best of our knowledge, this favonoid was the first apoptosis inducer to be identified in CLL cells [17] and is still one of the most potent. Flavopiridol acts by downregulating the antiapoptotic proteins Mcl-1 and XIAP through inhibition of cyclin-dependent kinases (CDK) required for RNA polymerase II activation [18]. Consequently, transcription of many short-lived proteins is decreased resulting in downregulation of key proteins such as Mcl-1, Bcl-xL, several IAP (XIAP, c-IAP-2, survivin), C-Myc (a transcriptional activator of Mcl-1), Mdm-2 (a p53 antagonist) and p21 [19]. Flavopiridol's effects on transcription are therefore not specific but do affect a large number of proteins. It is noteworthy that the decrease in Bcl-2 mRNA does not change Bcl-2 protein levels. The initial clinical trials of various favopiridol regimens in CLL gave disappointing results, probably because the favonoid is strongly bound by plasma proteins [20]. More successful outcomes were recorded with a pharmacologically-based dosing schedule in a Phase II trial on refractory and genetically high-risk CLL patients, providing more than 50% of responses and improvements in progression-free survival [21]. However, dose-escalation is difficult because of the lack of predictive markers for toxicity. Flavopiridol is currently investigated in combination with other agents and as a means of eradicating residual disease after chemotherapy [20].

#### Other CDK inhibitors

Other CDK inhibitors are capable of inducing CLL cell apoptosis via the inhibition of Mcl-1 transcription: they include the natural compound roscovitine/seliciclib (a starfish-derived alkaloid) [22] and the synthetic agents SNS-032 and dinaciclib [23,24]. The latter two agents are currently in clinical evaluation in CLL. Like flavopiridol, several other plant-derived flavones such as apigenin (Figure 2) are CDK inhibitors that induce apoptosis by transcriptional suppression of Mcl-1 [25]. This property might contribute to apigenin's proapoptotic effects on CLL cells [26], although other mechanisms also play important roles (see below).

#### Homoharringtonine and silvestrol

Homoharringtonine (Figure 2) and silvestrol are alkaloids isolated from Asian plants which were originally described as translation inhibitors. Both compounds are able to induce mitochondrial apoptosis in CLL cells by downregulating Mcl-1 [27,28]. Indeed, translational inhibition preferably targets labile proteins. However, a very recent report indicates that inhibition of Mcl-1 translation is not critical for the proapoptotic effects of the compounds and that they act via several different cell death mechanisms [29]. Silvestrol has shown *in vivo* antileukemic activity in the TCL-1 transgenic mouse model of human CLL [28]. Interestingly, sorafenib (a multikinase inhibitor) induces CLL cell death by translational downregulation of Mcl-1 [30].

#### Specific inhibitors of Bcl-2 family protein expression

Whereas CDK inhibitors and translational inhibitors do not selectively target antiapoptotic molecules, specific inhibition of Bcl-2 protein expression can be envisaged because Mcl-1 siRNA is capable of triggering CLL cell apoptosis *in vitro* [13]. However, technical obstacles currently prevent the RNA interference approach from being applied in the clinic [31]. The clinical development of short hairpin RNA mimicking endogenous microinterfering RNA (miR) appears to be even more difficult to apply [32] whereas the loss of miR-15a1 and miR-16 (targeting notably Bcl-2 and Mcl-1) in CLL was the first event of this type to be discovered [33]. In contrast, the antisense oligonucleotide strategy (targeting mRNA for degradation) has already been tested in the clinic with oblimersen (the most extensively tested Bcl-2 antisense oligonucleotide). Unfortunately, oblimersen has only moderate therapeutic activity in CLL patients even when combined with fludarabine and cyclophosphamide

[34]. This poor result was attributed to off-targets effects and so attempts to overcome the latter have been proposed recently [35].

### 2.2 Inhibitors of the functional activity of prosurvival Bcl-2 proteins

The BH3 mimetic concept prompts the development of small molecules capable of mimicking the BH3-only proteins which are natural, direct antagonists of the prosurvival Bcl-2 proteins. These small molecules are designed to bind to the prosurvival proteins (with the same high affinity as the natural ligands), inhibit the antiapoptotic activity of the latter (by releasing the sequestered proapoptotic Bax and Bak) and thus induce apoptosis [36]. The generated BH3 mimetics are either short peptides modeled on BH3 domains or small organic molecules (identified by screening natural product libraries or *in silico*-designed compounds for their ability to bind Bcl-2 proteins). However, many compounds thus identified did not fully meet the two main criteria defining an authentic BH3 mimetic, i.e. (i) high-affinity binding to the targets and (ii) induction of Bax and Bak-dependent apoptosis [36]. Indeed, these putative BH3 mimetics were found to act at least partly through off-target effects, such as the generation of reactive oxygen species (ROS), endoplasmic reticulum stress, induction of the BH3-only Noxa, caspase-independent or autophagic cell death [37–40].

#### The ABT series

The organic molecule ABT-737 (Figure 2) targets Bcl-2, Bcl-xL and Bcl-w (but not Mcl-1 or A1) and, for a time, was considered to be the sole *bona fide* BH3 mimetic [37]. Initially found to decrease the viability of CLL cells [37], ABT-737 was demonstrated to induce apoptosis through activation of the mitochondrial pathway [41]. Clinical trials have been developed with the orally available version ABT-263 (navitoclax): the recent published Phase I data are promising, with a partial response rate of 35% in relapsed or refractory CLL patients [42]. To avoid the dose-limiting thrombocytopenia associated with ABT-263 (due to inhibition of Bcl-xL activity), the ABT-199 derivative was designed to bind specifically to Bcl-2. This BH3 mimetic was found to reduce the tumor burden in the first three CLL patients recruited into an ongoing clinical trial [43].

#### Obatoclax

Obatoclax (GX15-070) is a synthetic derivative of natural prodigiosins (Figure 2). It can bind to all prosurvival Bcl-2 proteins albeit with low affinity [44]. This putative pan-BH3 mimetic, whose apoptotic activity has been attributed to various mechanisms (including Noxa induction and inhibition of AKT/mTOR pathway), was found to induce mitochondrial apoptosis in CLL patients' cells [45]. Recent data indicate that obatoclax can directly activate Bax (one of the two executioner molecules in mitochondrial membrane permeabilization) [46]. However, it is not known whether this activity occurs in CLL cells. In Phase I clinical trials, obatoclax have failed to demonstrate significant single agent efficacy in CLL. Ongoing Phase II studies will determine whether obatoclax might be active when combined with other drugs [47].

#### The gossypol family

Gossypol is a natural, polyphenolic aldehyde derived from cotton seed (Figure 2) that was already known for its proapoptotic activity prior to the discovery of its putative BH3 mimetic property [48]. Gossypol acts at least partly through off-target mechanisms such as ROS generation, Noxa induction, autophagic cell death [38,39,49,50]. It has been shown that gossypol induces mitochondrial apoptosis in CLL cells [51].The isomer AT-101 proved to be even more active and capable of overcoming stroma-mediated Mcl-1 induction and apoptosis prevention [52]. Phase I/II trials of AT-101 in CLL have indicated only limited single-agent therapeutic efficacy [36,53]. Several gossypol derivatives (acting predominantly through BH3 mimicry) are currently in preclinical studies [48]; these include apogossypolone (ApoG2) which was recently reported to induce apoptosis in CLL cells [54].

#### Compound 072RB

The peptide 072RB derived from the BH3 domain of Bim is another putative pan-BH3 mimetic that can induce CLL cell apoptosis and this effect is associated with Bcl-xL and Mcl-1 downregulation [55].

### 2.3 Upregulators of proapoptotic Bcl-2 proteins

#### Histone deacetylase inhibitors

Histone deacetylation is an epigenetic mechanism for transcriptional repression. Histone deacetylases (HDAC) are overexpressed in many cancers including CLL, in which they mediate epigenetic silencing of miR-15a, miR-16 and miR-29b [56]. A number of natural and synthetic HDAC inhibitors (HDACi) such as depsipeptide, vorinostat, LBH589, and MGCD0103 (see Figure 2) can promote different types of cell death in CLL cells notably extrinsic death receptor and intrinsic mitochondrial apoptosis [57–59]. Although ROS generation has been implicated in HDACi-induced apoptosis, it was reported that some HDACi act through transcriptional upregulation of Noxa and Bim mRNA [58]. Clinical data on HDACi in CLL were disappointing: depsipeptide and MGCD0103 have shown strong adverse effects and/or limited therapeutic benefit for patients [60,61]. This may be related to the fact that HDACi upregulate not only proapoptotic proteins but also Mcl-1 (mRNA and protein) in CLL cells [62]. Combinations with other drugs are currently under clinical evaluation.

#### Proteasome inhibitors

The proteasome complex catalyses the degradation of proteins targeted by ubiquitin and represents the major regulatory pathway in protein turnover [63]. Various classes of chemically distinct proteasome inhibitors have been characterized. They include both synthetic compounds such as the prototype bortezomib (Figure 2) and natural products such as lactacystin and epoxomicin. These compounds are found to affect key regulatory cellular processes and have antitumor functions by inducing apoptosis. Proteasome inhibitors enhance the stability of many proteins and cause their accumulation.

It is well established that two targets are IkBalpha (the physiologic inhibitor of NF-kB, which governs the induction of a number of antiapoptotic proteins) and the tumor suppressor p53 (which is known to activate the transcription of proapoptotic proteins). Two other targets, the BH3-only proteins Bim and Noxa, are increasingly thought to be crucial for the proteasome inhibitors' proapoptotic mechanisms of action [64].

More than a decade ago, the proteasome inhibitors lactacystin and MG-132 were found to be potent inducers of caspase-dependent mitochondrial apoptosis in CLL cells [65]. Bortezomib (which has been approved for the treatment of multiple myeloma and mantle cell lymphoma) also induces apoptosis in CLL cells by enhancing the stability and eliciting the accumulation of the BH3-only protein Noxa [66]. However, a clinical trial of bortezomib in CLL evidenced several toxic side-effects and failed to produce objective responses [67]. These disappointing results may well be related to the fact that bortezomib and other proteasome inhibitors (e.g. MG-132 and epoxomicin) also induce Mcl-1 accumulation notably in CLL cells: this would decrease the proteasome inhibitors' apoptotic response and thus therapeutic efficacy [68]. Interestingly, carfilzomib (a second-generation proteasome inhibitor) shows activity in CLL cells through an atypical mechanism, which has prompted the initiation of a Phase I clinical study [69]. However, carfilzomib also promotes Mcl-1 upregulation [70]. Combinations of certain proteasome inhibitors with other drugs are tested in ongoing clinical trials.

#### Plant-derived proteasome inhibitors

A variety of compounds present in fruits, vegetables and other plants can inhibit proteasome activities [63,71]. They include polyphenols such as resveratrol and curcumin, flavonoids (also belonging to the wide class of polyphenols) such as the flavone apigenin, the flavonol quercetin and the catechin epigallocatechin gallate (EGCG) as well as alkaloids of the terpenoid family such as the triterpen celastrol [72]. All these natural proteasome inhibitors display proapoptotic properties [71–73]. It is noteworthy that resveratrol, curcumin, apigenin, quercetin and EGCG induce CLL cell apoptosis (see below) as does the synthetic diaminomethoxyflavone [74] that we have recently shown to be a proteasome inhibitor [75]. It is therefore possible that these compounds activate CLL cell apoptosis at least partly through the inhibition of proteasomal degradation.

#### Hyperforin

Hyperforin is a phloroglucinol purified from the plant St John's wort (Figure 2). It has multiple biological properties, including *in vitro* and *in vivo* antitumor effects which are associated in some cases with apoptosis induction [76]. We were the first to show that hyperforin triggers caspase-dependent mitochondrial apoptosis in primary CLL cells [77]. This natural phloroglucinol acts through upregulation of the BH3-only protein Noxa, possibly via inhibition of proteasomal activity [78,79]. Interestingly, hyperforin-induced Noxa upregulation occurs without change in Mcl-1 expression [79].

#### Drugs that upregulate BH3-only proteins in CLL

Although DNA-damaging chemotherapy is thought to kill tumor cells mainly through the p53 pathway, the mechanisms involved have still not been unambiguously defined. A recent study demonstrated that the anticancer effect of cyclophosphamide in a transgenic mouse model of lymphoma requires not only the BH3-only proteins Puma and Noxa (which are p53 targets) but also the p53-independent Bim [80]. Whereas Puma strictly depends on p53, Noxa can also be regulated independently of p53. Many drugs used to treat CLL patients can trigger apoptosis *in vitro*; interestingly, this effect is associated with induction of at least one of the three proapoptotic BH3-only proteins mentioned above. Fludarabine can induce upregulation of Puma mRNA expression [81], and glucocorticoids trigger caspase-dependent apoptosis by increasing Bim mRNA levels [82]. Cisplatinium acts by Noxa upregulation as a result of ROS generation [83]. The purine nucleoside AICA-riboside (also called acadesine, see Figure 2) induces mitochondrial apoptosis of CLL cells via p53-independent increases in Noxa and Bim mRNA levels [84]. Acadesine is currently being evaluated in a Phase I/II trial in CLL. The purine analog and alkylating agent bendamustine (Figure 2) activates the mitochondrial apoptotic pathway by upregulating Puma and Noxa. Unlike Puma, the Noxa upregulation seems to result from ROS generation; bendamustine also induces caspase-independent cell death [85].

#### Other CLL cell apoptosis inducers upregulating BH3-only proteins

Aspirin was originally found to induce caspase-dependent apoptosis in CLL cells and this effect was recently associated with Noxa upregulation [86]. The synthetic phenylacetylenesulfonamide (PAS, also refered to as pifithrin-mu) exerts similar effects via a p53-independent increase in Noxa mRNA levels [87]. Another CLL cell apoptosis inducer, forodesine (a purine nucleoside phosphorylase inhibitor), can modulate a number of apoptosis proteins and stimulate Bim protein expression [88]. A pharmacodynamic study of oral forodesine has shown its biological activity in CLL patients [89].

### 2.4 Other natural CLL cell apoptosis inducers

#### Resveratrol, other grape-derived polyphenols and derivatives

Trans-resveratrol is a phytoalexin from the stilbene family of polyphenols (Figure 2) which is present in numerous plants, fruits and vine products. This compound was already known to protect against cardiovascular diseases. Extensive *in vitro* studies have revealed that resveratrol also displays potential anticancer properties. The polyphenol regulates many different molecular targets and signaling pathways [90]. These include p53, AP-1, members of the IAP and Bcl-2 families (such as Puma, Bim and Noxa), proteasome activity, ROS production, NF-kB, PI3K/AKT, MAP kinase, TRAIL-death receptor and mitochondrial pathways. Resveratrol therefore displays the hallmarks of a multi-target apoptosis inducer. We were the first to report that resveratrol induces apoptosis in primary CLL cells, triggers the mitochondrial pathway and activates caspase-3 [91]. Similar effects have been observed with other wine-derived polyphenols (vineatrols) and an acetate derivative of epsilon-viniferin (a resveratrol dimer) [74,92]

#### Curcumin

Curcumin (an active component of turmeric) is another well-known polyphenol with a broad range of biological properties, including the ability to induce apoptosis in CLL cells [93]. Although it was originally suggested that curcumin acts through NF-kB inhibition, the polyphenol also inhibits proteasome activity, AKT and other signaling pathways (e.g. STAT3), downregulates Mcl-1 and XIAP and upregulates Bim [93].

#### Epigallocatechin gallate

This catechin (or flavanol) is the most active flavonoid in green tea (Figure 2). Epigallocatechin gallate (EGCG) has pleiotropic effects. It can notably trigger caspase-dependent mitochondrial apoptosis in many tumor models, including primary CLL cells in which it downregulates Mcl-1 and XIAP [94]. Several other crucial apoptotic pathways such as proteasome inhibition are involved in EGCG's mechanisms of action [73]. A Phase II study of EGCG in CLL has shown promising results with reduction in lymphocytosis and/or adenopathy in 29 out of 42 patients (69%) [95].

#### Quercetingallate

Like EGCG, this tea-derived flavonol (Figure 2) is a multi-target apoptosis inducer and proteasome inhibitor [71]. Quercetin can trigger both the extrinsic and intrinsic pathways in primary CLL cells, notably by Mcl-1 downregulation; the mechanism involves inhibition of the PI3K/AKT pathway, which in turn leads to the instability Mcl-1 mRNA and protein [96,97].

#### Apigenin and diaminomethoxyflavone

The natural flavone apigenin (Figure 2) has proapoptotic activity in CLL cells, and this effect has been associated with decreased AKT phosphorylation and inhibition of the PI3K/AKT pathway [26]. Given that apigenin is known to inhibit both proteasome and CDK activities [25,71] and to target several apoptotic pathways [98], the compound may act through several mechanisms in CLL cells. As mentioned above, diaminomethoxyflavone is an apoptosis inducer in CLL cells [74] and this synthetic flavone can also inhibit the PI3K/AKT pathway and proteasome activity [75].

#### Xanthohumol

This plant-derived, prenylated flavonoid is able to induce CLL cell death through caspase-dependent apoptosis resulting from activation of endoplasmic reticulum stress [99]. In the same study, xanthohumol was found to inhibit both proteasome activity and Mcl-1 expression and these effects were associated with inhibition of the translation initiator factor eIF2alpha but not the modulation of NF-kB activation. Hence, xanthohumol appears to be a natural proteasome inhibitor capable of downregulating Mcl-1 in CLL cells.

#### Triterpenoids

Natural and synthetic triterpenoids are potential antitumor compounds possessing multifunctional properties including antiproliferative, antiangiogenic, antiinflammatory and proapoptotic activities. The synthetic triterpenoid 2-cyano-3,12-dioxoolean-1,9-dien-28oic acid (CDDO, see Figure 2) was shown to elicit apoptosis via the mitochondrial pathway in CLL cells [100] and exert antileukemic effects in a transgenic mouse model of CLL [101]. Unlike the natural triterpen celastrol, CDDO is not a proteasome inhibitor but reportedly acts through various mechanisms, including inhibition of the NF-kB pathway, endoplasmic reticulum stress, death receptor and mitochondrial pathways.

#### Xanthones

Xanthones were originally isolated from the tropical fruit mangosteen. They are known to possess a wide spectrum of pharmacological properties and anticancer effects. The compounds do modulate various targets and signaling pathways (including apoptosis pathways) [102]. We have shown for the first time that several xanthones purified from African trees (e.g. allanxanthone C, shown in Figure 2) can induce caspase-dependent apoptosis in CLL cells via activation of the mitochondrial pathway or other mechanisms [103]. We further reported that allanxanthone C and macluraxanthone have *in vivo* therapeutic effects in a xenograft murine model of CLL [104].

#### Arylcoumarin analogs of combretastatins

Combretastatins are natural stilbenoid polyphenols isolated from African trees. They have antimitotic properties by binding to tubulin, inhibiting its polymerization and thus blocking microtubule assembly. It was reported that the mitotic catastrophe triggered by some combretastatins in human leukemia cells is associated with caspase-dependent apoptosis [105]. We have shown that arylcoumarin derivatives, analogs of combretastatin-A4 (that were synthesized to avoid isomerization into the inactive trans-configuration) can induce the caspase-dependent mitochondrial pathway of apoptosis in CLL cells [106].

#### Vinblastine

The mitotic spindle poison vinblastine was the first vinca alkaloid used in the treatment of CLL. It was recently shown that vinblastine induces acute apoptosis in CLL cells (independently of cell cycle) and that this effect is associated with an increase in Noxa transcripts [107]. Other microtubule-disrupting agents (combretastatin-A4, vincristine and vinorelbine) have the same effects [107].

### 2.5 Other types of CLL apoptosis inducers

#### Fenretinide

The synthetic retinoid N-(4-hydroxyphenyl) retinamide (called fenretinide) promotes the intrinsic apoptotic pathway in CLL cells via ROS generation. This is accompanied by Mcl-1 protein degradation resulting from jun N-terminal kinase (JNK) activation [108]. Two other mechanisms have been suggested: inactivation of NF-kB and upregulation of Noxa (which is known to target Mcl-1 for proteasomal degradation).

#### IAP antagonists

Two strategies to antagonize caspase inhibitors of the IAP family have been developed [109]. Firstly, small molecules mimicking Smac (the physiological antagonist of IAP proteins) were designed: a Smac mimetic antagonizing XIAP was found to enhance TRAIL-induced CLL cell apoptosis [10]. Secondly, the antisense strategy has been tested in a clinical trial of an XIAP antisense oligonucleotide (AEG35156) in CLL, but the results were not encouraging.

#### Inhibitors of various kinases and signaling pathways

A number of agents are currently tested in clinical trials on the basis of their ability to indirectly induce CLL cell apoptosis [5,8,16]. This is the case for CAL-101 (PI3K inhibitor), Akti-1/2 and A-443654 (AKT inhibitors), BAY117082 (NF-kB inhibitor), inhibitors of kinases involved in BCR signaling such as dasatinib (Lyn kinase) or fostamatinib (Syk) and other kinase inhibitors such as sorafenib (multikinase) or imatinib (c-Abl kinase). Furthermore, activated heat-shock protein 90 (Hsp90) is a chaperone molecule for the zeta-associated protein ZAP-70 which positively regulates BCR signaling and is expressed by CLL cells from patients with aggressive disease. Inhibitors of Hsp90 (e.g. 17-DMAG) induce ZAP-70 degradation and apoptosis of CLL cells and are tested in ongoing clinical trials in CLL.

##### Interleukin-21 (IL-21)

This cytokine has been reported to mediate CLL cell death through upregulation of the BH3-only protein Bim [110].

## 3 COMMENTS AND DISCUSSION

### 3.1 The structural and functional diversity of CLL cell apoptosis inducers

Inducers of CLL cell apoptosis display broad functional diversity (Table 1). The class encompasses inhibitors of transcription (CDK inhibitors), translation, proteasome activity (affecting protein turnover), HDACs (affecting epigenetic regulation), various kinases (affecting different signaling pathways) and the antiapoptotic activity of Bcl-2 proteins (BH3 mimetics). They also include transcription modulators, cytokines, corticoids and various types of cancer drugs (nucleotide analogs, alkylating agents and microtubule-disrupting agents).

Apoptosis inducers also display structural diversity (Table 2). Many are natural compounds (generally derived from plants) or their semisynthetic/synthetic derivatives. This group includes some of the most active compounds such as the prototypic flavopiridol. Although many plant-derived apoptosis inducers are flavonoids (flavones, EGCG, quercetin, xanthohumol) or other types of polyphenols (gossypol, resveratrol, curcumin), other families are also represented, such as xanthones, phloroglucinols (hyperforin) and various alkaloids (homoharringtonine, silvestrol). Some apoptosis inducers are derived from bacteria (depsipetide, lactacystin). Furthermore, certain cancer drugs (vinblastine for example) are themselves of natural origin. There are also many synthetic apoptosis inducers including BH3 mimetics (the ABT series), triterpens (CDDO), various enzyme inhibitors (proteasome, HDACs, kinases and survival pathways), antisense oligonucleotides and cytostatic drugs.

### 3.2 The mechanisms of action of apoptosis inducers

**Figure 3:**
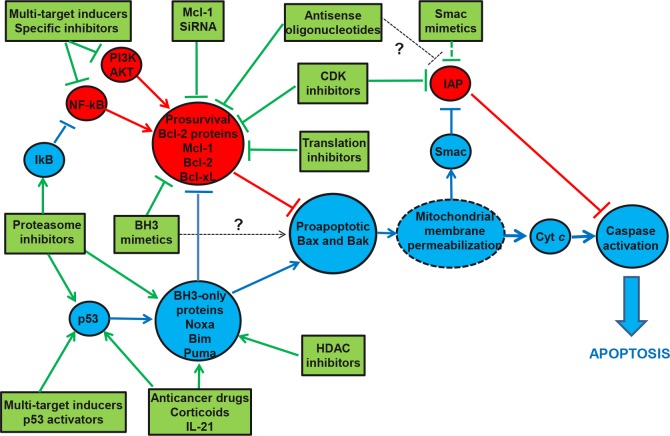
Mechanisms of action of apoptosis inducers that activate the mitochondrial caspase-dependent pathway in CLL cells Apoptosis inducers can either inhibit antiapoptotic proteins or stimulate proapoptotic proteins. Their main targets are Bcl-2 family members. In the former mechanism, several types of compounds downregulate the expression of prosurvival Bcl-2 proteins whereas BH3 mimetics inhibit their activity (via sequestration of Bax and Bak, the executioner molecules in the mitochondrial membrane permeabilization). In the latter mechanism, apoptosis inducers upregulate the expression of BH3-only proteins (the endogenous antagonistic ligands for prosurvival Bcl-2 proteins, some of which are able to activate Bax and Bak directly). Inhibition of antiapoptotic factors and stimulation of proapoptotic proteins can also be achieved indirectly by some compounds including multi-targets apoptosis inducers, which are capable of interfering with various signaling pathways (e.g., NF-kB, PI3K/AKT, p53). Inhibition of other antiapoptotic proteins such as IAP (antagonizinz caspase activity) is not sufficient to trigger apoptosis but does amplify the effects of certain apoptosis inducers. Blue circles: proapoptotic factors; red circles: antiapoptotic molecules; green boxes: apoptotic inducers. NF-kB, nuclear factor-kappaB; PI3K, phosphoinositol-3 kinase; SiRNA, small interfering RNA; CDK, cyclin-dependent kinase; HDAC, histone deacetylase; IL-21, interleukin-21; IAP, inhibitor of apoptosis protein; Cyt c, cytochrome c; Smac, second mitochondria-derived activator of caspases.

As appears from the data, apoptosis inducers act via two major mechanisms: (i) inhibition of either the expression or activity of antiapoptotic proteins and (ii) upregulation of proapoptotic molecules (Figure 3). The targets are mostly Bcl-2 family proteins; this may involve either downregulation of the family's prosurvival members (mainly Bcl-2 and Mcl-1), which are crucial players in the impaired apoptosis in CLL, or upregulation the BH3-only proteins (Bim, Puma, Noxa), which are functional antagonists of the prosurvival members. These data have therefore validated the Bcl-2 family proteins to be crucial targets for novel therapeutic strategies, which prompted the development of BH3 mimetics. Regarding the inhibition of IAP family proteins, it seems to require TRAIL-activation of the death receptor pathway for the full induction of apoptosis. Apoptosis inducers can also act indirectly through either inactivation of NF-kB or PI3K/AKT survival pathways (which are responsible for transcriptional activation of Bcl-2 and IAP proteins) or stimulation of the proapoptotic p53 pathway (resulting in induction of BH3-only proteins). Lastly, some multi-target apoptosis inducers (such as resveratrol, curcumin, flavonoids, xanthones, hyperforin and triterpens) are capable of acting through several of these pathways (NF-kB, PI3K/AKT and p53). Most of these compounds are natural proteasome inhibitors.

The fact that many apoptosis inducers directly target Bcl-2 family proteins constitutes a theoretical advantage over chemotherapeutic agents, since these proteins regulate the initial molecular events responsible for triggering the caspase-dependent mitochondrial apoptotic pathway. Indeed, apoptosis inducers have mostly been found to trigger mitochondrial membrane permeabilization, cytochrome *c* release and the caspase cascade. Nevertheless, apoptosis inducers can also use other mechanisms: caspase activation can result from endoplasmic reticulum stress, ROS generation or the death receptor pathway, and some compounds can even trigger caspase-independent apoptosis or autophagic cell death (Table 3).

**Table 3:** Pathways other than the intrinsic mitonchondrial pathway used by apoptosis inducers in chronic lymphocytic leukemia cells

**Cell death pathways**	
Extrinsic cell death receptor pathway	
Endoplasmic reticulum stress response	
Reactive oxygen species (ROS) generation	
Caspase-independent apoptosis	
Autophagic cell death	
p53 pathway	
**Survival pathways**	
Nuclear factor-kappaB (NF-kB)	
Phosphatidylinositol-3 kinase (PI3K)	
Protein kinase B (AKT)	
Mammalian target of rapamycin (mTOR)	
**Other signaling pathways**	
B cell receptor	
Hsp90	
Jun N-terminal kinase (JNK)	
Mitogen activated protein (MAP) kinase	

### 3.3 On the clinical use of apoptotic inducers in CLL

The most encouraging clinical data have been obtained with flavopiridol (the semisynthetic CDK inhibitor that decreases Mcl-1 transcription) and ABT-263 (the BH3 mimetic that antagonizes the activity of prosurvival Bcl-2 proteins). Promising results were also observed recently with the natural flavonoid EGCG, which inhibits both the proteasome and Mcl-1 expression. Extensive ongoing trials are assessing other CDK inhibitors (SNS-032, dinaciclib), other BH3 mimetics (the new ABT-199, gossypol derivatives), translation inhibitors (homoharringtonine), sorafenib and other kinase inhibitors, bendamustine, forodesine, acadesine and Hsp90 inhibitors. Some synthetic apoptosis inducers with high potency *in vitro* have turned out to be disappointing when used to treat CLL patients: this is the case of the proteasome inhibitor bortezomib, several HDAC inhibitors (MGCD0103 for instance) and antisense oligonucleotides (targeting Bcl-2 or XIAP). Likewise, the BH3 mimetics obatoclax and AT-101 have not shown therapeutic benefits in CLL. Apoptosis inducers might be more successful in the clinic when rationally combined with conventional chemo-immunotherapy. Hence, flavopiridol, obatoclax, proteasome inhibitors and HDAC inhibitors are currently being tested in combination with other drugs. The triterpen CDDO has not yet been evaluated in CLL patients despite its marked therapeutic effects in a transgenic mouse model of CLL.

Only a few natural apoptotic inducers have been clinically evaluated in CLL. Some plant-derived compounds deserve to be tested in the clinic such as hyperforin. Indeed, the latter advantageously upregulates expression of the proapoptotic protein Noxa but not that of the antiapoptotic Mcl-1 (as compared to bortezomib and HDAC inhibitors which upregulate both of these proteins). Several improved derivatives of hyperforin such as aristoforin have shown innocuousness and efficacy in animal models [76]. Allanxanthone B and macluraxanthone also appear to have therapeutic potential since they were found to exhibit antileukemic effects in a xenograft mouse model of CLL [104]. Lastly, it would be interesting to evaluate natural flavonoids such as apigenin (which inhibits both the proteasome and CDKs) and other plant-derived proteasome inhibitors also capable of Mcl-1 downregulation (e.g. quercetin).

However, the main concern with natural apoptosis inducers is that they often affect multiple molecular targets and signaling pathways; this is the case for resveratrol, curcumin, xanthones and hyperforin, for instance. It is therefore difficult to determine which mechanism is responsible for the proapoptotic effects. Moreover, this pleiotropy may result in adverse drug reaction especially during dose escalation. It is noteworthy that this shortcoming also applies to synthetic apoptosis inducers that are not specific for a unique molecular target (e.g. CDK, proteasome and HDAC inhibitors). Likewise, a survival pathway inhibitor usually affects several other signaling pathways. This is even a problem with BH3 mimetics: (i) most putative mimetics act through off-target effects and (ii) the *bona fide* BH3 mimetic ABT-263 antagonizes both Bcl-2 and Bcl-xL, so that inhibition of Bcl-xL activity results in toxic thrombocytopenia in patients. In contrast, the newly developed ABT-199 antagonizes Bcl-2 but not Bcl-xL. All these data support the rational use of apoptosis inducers in combination therapy protocols. Furthermore, it is not surprising that BH3 mimetics such as ABT-737/263 and Bcl-2 antagonists in general also have antiangiogenic properties [111] since Bcl-2 is known to have proangiogenic activities. This may be of value inasmuch as (i) CLL cells constitutively secrete the angiogenic factors VEGF and bFGF and (ii) neo-angiogenesis is an important problem in CLL. Nevertheless, the characterization of apoptosis inducers and their mechanisms of action have enabled the identification of specific molecular targets with potential therapeutic interest as discussed below.

### 3.4 The Noxa/Mcl-1 axis as a critical drug target for apoptosis-based strategies

A number of apoptosis inducers that upregulate Noxa in CLL cells have been mentioned above: bortezomib and other proteasome inhibitors, several HDAC inhibitors, hyperforin, acadesine, bendamustine, cisplatinium, vinblastine and other vinca alkaloids, PAS and aspirin. Noxa upregulation was also found to be associated with apoptosis elicited by some agents whose proapoptotic effects on CLL cells have been described elsewhere (e.g. obatoclax, gossypol and derivatives and resveratrol). Furthermore, several compounds known for their proapoptotic activities on tumor cells other than CLL are capable of activating Noxa: this is the case for tetramethoxystilbene (a resveratrol analog), isoliquiritigenin (a plant-derived flavonoid), phenoxodiol (a semisynthetic isoflavone) as well as celastrol (a triterpen proteasome inhibitor) [112–115]. Lastly, it must be borne in mind that Noxa can be upregulated by the inhibition of proteasomal activities and that some flavonoids capable of inducing apoptosis in CLL cells have also been characterized as natural proteasome inhibitors (EGCG, quercetin, apigenin). Given that Noxa can bind to Mcl-1 but not other survival Bcl-2 proteins and that Mcl-1 clearly has a critical antiapoptotic role, the Noxa/Mcl-1 axis is an attractive target for apoptosis-based strategies in CLL [116,117].

### 3.5 Novel developments in apoptosis-targeted anti-CLL therapies

A number of reports have suggested combining flavopiridol or other Mcl-1 downregulators with proteasome or HDAC inhibitors (both of which enhance Noxa and Mcl-1 expression) or ABT-263 (which does not antagonize Mcl-1). It is noteworthy that several flavonoids both downregulates Mcl-1 through various different mechanisms (inhibition of CDKs and transcription, translation and the PI3K/AKT pathway, for example) and inhibits the proteasome in CLL cells: these include xanthohumol, apigenin, EGCG and quercetin. Diaminomethoxyflavone is also an apoptosis inducer in CLL cells that can inhibit the PI3K/AKT pathway and proteasome activity. Since proteasome inhibition is known to result in the stabilization and accumulation of the BH3-only protein Noxa, it would be particularly interesting to determine whether some of the natural proteasome inhibitors can effectively enhance Noxa expression in CLL cells. Their chemical structure might then serve as a framework for the design of more efficient derivatives. Likewise, other polyphenols that are multi-target apoptosis inducers in CLL cells (such as resveratrol) are already known to enhance Noxa and inhibit Mcl-1 (notably through NF-kB inhibition). Moreover, Noxa expression (induced by ROS generation, for instance) can lead to proteasomal degradation of Mcl-1; this mechanism has been described for the retinoid derivative fenretinide.

Some flavonoids show a structural resemblance to gossypol (see Figure 2) which is thought to function as a BH3 mimetic. It would therefore be useful to establish whether natural apoptosis inducers other than gossypol are capable of BH3 mimicry. In such a case, their chemical structures might serve as a framework for designing new BH3 mimetics.

The development of BH3 mimetics capable of mimicking Noxa and specifically antagonizing Mcl-1 has also been considered. Proof of concept for Noxa-like BH3 mimetics has been provided by the characterization of two compounds: a variant of the BH3 domain of Bim (called Bim__s__2A) and a stabilized alpha-helix of Bcl-2 domains (SAHB) derived from the BH3 region of Mcl-1 itself (called Mcl-1 SAHB) [50]. Furthermore, a prototypical Mcl-1-specific BH3 mimetic (MIM 1) has recently been identified [118]. This small molecule displaces Mcl-1 SAHB from its binding partner Mcl-1 and induces apoptosis through Bax/Bak activation in Mcl-1-dependent leukemia cells. Moreover, the first pan-BH3 mimetic has been discovered: it is a SAHB modeled on the Bim BH3 domain (called Bim SAHB) that (i) targets a broad range of survival Bcl-2 proteins (Bcl-xL, Bcl-w, Mcl-1 and Al), (ii) induces caspase-dependent mitochondrial apoptosis in leukemia/lymphoma cells that are resistant to ABT-737 and (iii) suppresses tumor growth in a mouse model of acute myeloid leukemia [119]. Lastly, the BH3 mimetic concept has been used to design a compound capable of mimicking an “activator” BH3-only protein and this compound has led to the characterization of the direct Bax activator molecule BAM7 [120]. It remains to be seen whether the new BH3 mimetics MIM 1, Bim SAHB and BAM7 are capable of inducing apoptosis in primary CLL cells.

## 4 CONCLUSIONS

Attempts to reactivate the apoptotic machinery in CLL cells *in vitro* have not yet led to the discovery of novel therapeutics capable of curing CLL. However, a number of the identified apoptosis inducers might be used to improve the treatment of CLL patients via combination with conventional chemo-immunotherapy. Moreover, some natural apoptosis inducers deserve to be tested in the clinic. Others might constitute the framework for designing more efficient derivatives. The major drawback of most apoptosis inducers is that they do not have specific molecular targets. Nevertheless, the characterization of the inducers' mechanisms of action has validated the Bcl-2 family members and the Mcl-1/Noxa axis in particular as potential therapeutic targets in CLL. The BH3 mimetic concept is a potent weapon for circumventing the poor specificity of many apoptosis inducers. The discovery of new types of BH3 mimetics (ABT-199, MIM 1, Bim SAHB and BAM7) constitutes a critical step forward in the development of novel, apoptosis-targeting drugs for the treatment of CLL and other cancers.
